# Data Gap: Air Quality Networks Miss Air Pollution from Concentrated Animal Feeding Operations

**DOI:** 10.1021/acs.est.3c06947

**Published:** 2023-11-30

**Authors:** Alyssa M. Burns, Gabriel Chandler, Kira J. Dunham, Annmarie G. Carlton

**Affiliations:** † Department of Chemistry, University of California, Irvine, California 92617, United States; ‡ Department of Mathematics and Statistics, Pomona College, Claremont, California 91711, United States; § Food and Water Watch, Washington, District of Columbia 20036, United States

**Keywords:** agriculture, ammonia, livestock waste, air quality

## Abstract

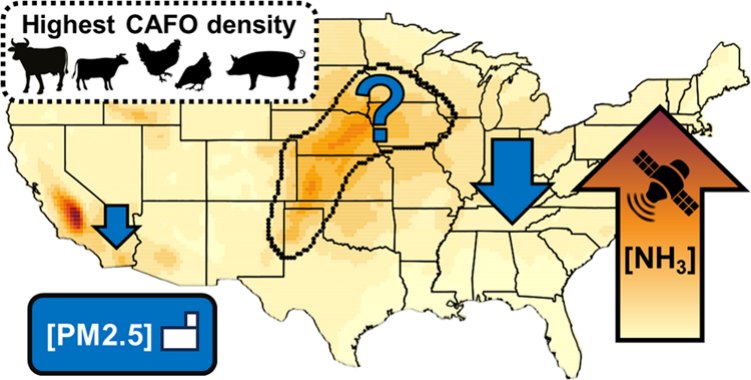

In the U.S., the agricultural sector is the largest controllable source of several air pollutants, including ammonia (NH_3_), which is a key precursor to PM_2.5_ formation. Livestock waste is the dominant contributor to ammonia emissions. In contrast to most controllable air pollutants, satellite records show ammonia mixing ratios are rising. The number of confined animal feeding operations (CAFOs) that generate considerable livestock waste is also increasing. Spatial and temporal trends in USDA-reported animal numbers normalized by county area at medium and large CAFOs provide plausible explanations for patterns in satellite-derived NH_3_ over the contiguous U.S. (CONUS). The correlation between summertime ammonia derived from the European Space Agency’s (ESA) Infrared Atmospheric Sounding Interferometer (IASI) and CAFO animal unit density in 2017 is positive and significant (*r* = 0.642; *p* ≈ 0). The temporal changes from 2002 to 2017 in animal unit density and NH_3_ derived from NASA’s Atmospheric Infrared Sounder (AIRS) are spatially similar. Trends and ambient concentrations of PM_2.5_ mass in agricultural regions are difficult to assess relative to those of urban population centers given the sparseness of rural monitors in regulatory surface networks. Results suggest that in agricultural areas where ammonia concentrations and animal density are highest, air quality improvement lags behind the national average.

## Abstract 2

This is the first quantitative positive correlation between USDA Census of Agriculture animal units and remotely sensed ammonia.

## 1 Introduction

The U.S. Environmental Protection Agency (EPA) reports that agriculture is the largest source of anthropogenic emissions of ammonia (NH_3_), nitrous oxide (N_2_O), and methane (CH_4_).^1,2^ Emissions of nitric oxides (NO_*x*_; NO_*x*_ = NO + NO_2_), a variety of organic species and reduced sulfur compounds, and primary PM_2.5_ from agriculture are also documented.^3−6^ These emissions adversely affect air quality and ecological welfare, destroy stratospheric ozone, and add to the global burden of greenhouse gases.^2,7−17^

Domestically, U.S. agricultural ammonia emissions are estimated to cause over 12,000 premature deaths and incur societal costs of roughly $160 billion each year, primarily through the formation of fine particulate matter (PM_2.5_).^18,19^ As the most abundant basic gas in the atmosphere, ammonia readily reacts with oxidation products of sulfur dioxide (SO_2_) and NO_*x*_ to form ammonium sulfate and ammonium nitrate, which contribute substantially to ambient PM_2.5_, a criteria air pollutant (CAP). A 42% reduction in PM_2.5_ mass concentrations is observed over the contiguous U.S. (CONUS) from 2000 to 2022.^20^ The national average is weighted by monitoring site locations and driven largely by trends in the Northeast (73 sites, −48%), Ohio Valley (59 sites, −51%), and Southeast (63 sites, −48%). Decreased PM_2.5_ mass concentrations can be attributed to environmental policies, such as acid rain and ozone mitigation. For example, SO_2_ and NO_2_ are CAPs regulated through the National Ambient Air Quality Standards (NAAQS) framework and performance standards at controlled sources. Measurements at approximately 500 air quality monitoring locations^21,22^ document sharp reductions in ambient mixing ratios of SO_2_ and NO_2_ that contribute to declines in PM_2.5_ mass concentrations due to reduced formation of ammonium sulfate and ammonium nitrate, respectively.^23−25^ In sharp contrast and despite its similar contribution to PM_2.5_, there is no NAAQS for ammonia, and ambient concentrations are not as routinely monitored in regulatory networks. Multiple satellite products indicate that the atmospheric burden of NH_3_ is increasing over the U.S., most notably in agricultural regions.^26,27^

Atmospheric mixing ratios of ammonia are spatially heterogeneous and generally spike downwind in close proximity of sources due to the relatively short atmospheric lifetime (<1 day).^28−31^ Physical loss mechanisms such as wet and dry deposition dominate over chemical loss.^32^ Nitrogen deposition is increasing at National Atmospheric Deposition Program (NADP) monitoring locations influenced by agriculture.^15,28,29^ Similarly, since 2007, biweekly passive surface measurements from the Ammonia Monitoring Network (AMoN) document the highest ammonia concentrations are generally associated with agricultural locations.^33^ The number of agricultural operations with large numbers of feedlot-confined animals, commonly called concentrated animal feeding operations (CAFOs), is increasing. The Government Accountability Office (GAO) estimates that the number of CAFOs increased 230% from 1982 to 2002,^34^ in part due to economic factors that encourage increased agricultural intensity and higher animal density with the shift in market focus to exports.^35^ More recently, EPA data shows the number of CAFOs increased 16% from 2011 to 2022.^36^

Despite the growing number of CAFOs and their contribution to environmental pollution, accurate understanding of the number, size, and exact location of animals housed in CAFOs is difficult to acquire^34^ because consistent, public CAFO data is sparse. For example, some agricultural enterprises, including large-scale CAFOs, are not included in the USDA’s Census of Agriculture (hereafter, “AgCensus”) and other public data sets, due to USDA’s programmatic privacy concerns.^37^ CAFOs that meet certain definitions in the Clean Water Act are subject to the National Pollution Discharge Elimination System (NPDES) program, which regulates the discharge of pollutants from point sources to navigable waters of the U.S.^36^ EPA records NPDES permit data, but not all CAFOs discharge to navigable waters. The precise locations and animal populations of all CAFOs are not fully disclosed in any public inventory. Both the USDA and EPA data sets represent lower bounds for the actual number of CAFO-associated animals. CAFOs are more common in high-poverty and majority-non-White communities,^38,39^ and their pollution may represent an environmental justice issue.

In this work, we investigate animal density at CAFOs in the CONUS over a 15-year period (2002–2017) in relation to satellite-detected ammonia and surface level PM_2.5_ mass. We examine animal density at CAFOs to the extent possible with public data sets through analyses of the USDA Agricultural Census and EPA NPDES permit filings. We assess remotely detected ammonia and the evolving chemical climatology of rural areas in the context of surface measurements of PM_2.5_ mass and its chemical constituents. We apply analyses according to the EPA and USDA’s regional categories of the CONUS to explore the ability of current governmental accounting systems to accurately describe ambient air quality where ammonia pollution is greatest.

## 2 Materials and Methods

For all analyses, we investigate trends in the U.S. using various public, government-hosted data sets, as identified below. R statistical software is used for all data processing.^40^

### 2.1 County-Level Animal Density Maps and CAFO Trends

Under the Clean Water Act (CWA), one animal unit (AU) is equivalent to 1000 lb of live weight, which is roughly equivalent to 1 individual cow or cattle, 2.5 swine, and/or 30–125 egg-laying or broiler chickens depending on the manure management system.^41^ We employ the USDA Census of Agriculture reports, available at the county level every five years from 2002 to 2017.^37^ We focus on medium- and large-scale operations that meet the following descriptions: 500+ cattle/cows, 1000+ hogs, 500,000+ broilers sold annually, or 100,000+ egg layers. To normalize emissions among species, AUs are used instead of individual head counts. In this work, we define AUs more consistent with the USDA’s Economic Research Service (ERS) definitions, which account for animal lifetimes by employing “animal unit months”. One AU is approximately equivalent to 1.14 beef cattle, 0.74 dairy cows, 9.09 hogs, 250 laying hens, or 455 broilers.^42^ Other animal species are also industrially farmed,^43^ but are not assessed here. USDA may not fully disclose animal counts at the county level due to facility privacy concerns. We use state-level animal totals to estimate the number of undisclosed CAFO animal units in the AgCensus and assign those animals to CAFO facilities located in omitted counties. Animal data from the AgCensus are linked to county-specific land areas from USDA’s ERS using the Census Service’s unique Geographic Identifier (GeoID) for each county. AU density is calculated as the number of animals in each category normalized to AU definitions and divided by the county land area. Ordinary least-squares linear regression is used to examine trends in individual counties over time. Trends are defined as the slope of the regression lines (AU km^–2^ year^–1^).

### 2.2 Satellite Retrievals

Two satellite products are used to examine the spatial distributions and trends in ambient NH_3_. We focus on summertime values (meteorological summer defined as June, July, and August), as higher temperatures favor gas-phase NH_3_. The European Space Agency’s (ESA) Infrared Atmospheric Sounding Interferometer (IASI) provides NH_3_ observations with pixel size of 12 km in diameter at nadir and was recently evaluated with aircraft measurements over the U.S.^44^ NH_3_ values are averages over the vertical extent. The National Aeronautics and Space Administration (NASA) Atmospheric Infrared Sounder (AIRS) provides observations with a spatial resolution of 13.5 km at nadir,^45^ and NH_3_ values are taken at 918 hPa.^46^ We employ version 3.1.0 of the level 2 IASI files to examine the spatial extent of NH_3_ in 2017, the latest Census of Agriculture. AIRS data are available earlier in the record for the AgCensus and we employ this product to examine the annual trends from 2002 to 2017. To evaluate trends, we examine every grid point in the AIRS data set for which data existed for a minimum of five years in the available time window of the USDA censuses. We employ Pearson correlation to link variability between AUs and remotely sensed NH_3_ in 2017. We use ordinary least-squares to calculate linear fits to temporal trends and report trends as the slope of the regression line (ppb year^–1^).

### 2.3 Surface Air Quality Data and Data Set Synthesis

We employ air quality concentration and emission data from EPA public repositories. Ammonia emissions estimates are derived from the National Emissions Inventory (NEI), available every 3 years.^2^ We focus on 2017 emissions estimates, as the methodology for NH_3_ emissions from livestock waste changed substantially between the 2014 and 2017 inventories.^47^ The NEI identifies more than 50 sectors that contribute to NH_3_ emissions. We consolidate these sectors into 7 categories and keep the agricultural subsectors of fertilizer application and livestock waste separate. Ambient concentrations of PM_2.5_ mass, chemical constituents (NH_4_, NO_3_, SO_4_, and OC), and precursor gases (SO_2_ and NO_2_) are retrieved from EPA’s Air Quality System (AQS) pregenerated data files for all site and monitor locations that operated continuously from 2002 to 2017.^48^ We use all reported values, including Federal Reference Methods (FRM) and Federal Equivalency Methods (FEM). Measurements flagged in the AQS as exceptional events are removed. Total organic carbon values are converted to organic matter (OM) using the standard multiplier of 1.8 to better compare to other species in terms of mass concentrations.^49^ Similar to trends in animal density and satellite NH_3_, a linear regression is used to examine trends at individual monitor locations. Trends are defined as the slope of the regression line divided by the median concentration of the study time period (2002–2017), multiplied by 100% (% year^–1^).

Air quality trends are examined for both individual monitor locations and, more broadly, in regional analyses. Regions are informed by the EPA and USDA definitions. EPA commonly uses 9 U.S. Climate regions to describe PM_2.5_ trends.^50^ USDA uses Farm Production Expenditure Regions to examine the costs incurred running a farm, and groups states with consideration of farm output and size.^51,52^ Any state listed as an “estimate” for the USDA regions was included in the respective region. Employing either the EPA or USDA definitions, each region has a minimum of 10 PM_2.5_ continuous sites reporting over the study period (2002–2017) and a minimum of three speciation sites. Monitor coverage is the most sparse for ammonium ion measurements. We have more confidence in trends of PM_2.5_ mass and chemical constituents for regions where monitor density is highest (Tables S1–S4).

To more closely assess trends where both AU density and ammonia mixing ratios are relatively high and rising in the Midwest, we created a “hotspot” region for the year 2017. CAFO information from AgCensus is only available for unevenly spaced county-level designations. Consequently, we associate each AU value with the corresponding county centroid. A kernel smoother is applied to this data to obtain animal density estimates over the entire CONUS. We perform an identical smoothing technique to the IASI NH_3_ measurements. Though higher resolutions are possible with the IASI data,^53^ we apply this estimate for a consistent comparison with the spatially limited animal data. A region of high AU density is identified via a regression level set estimate (i.e., the coordinates where the estimated AU density exceeds a given value). We represent this hotspot as the boundary of the level set corresponding to the 91st percentile of the AU density across the CONUS.

## 3 Results and Discussion

**Figure 1:**
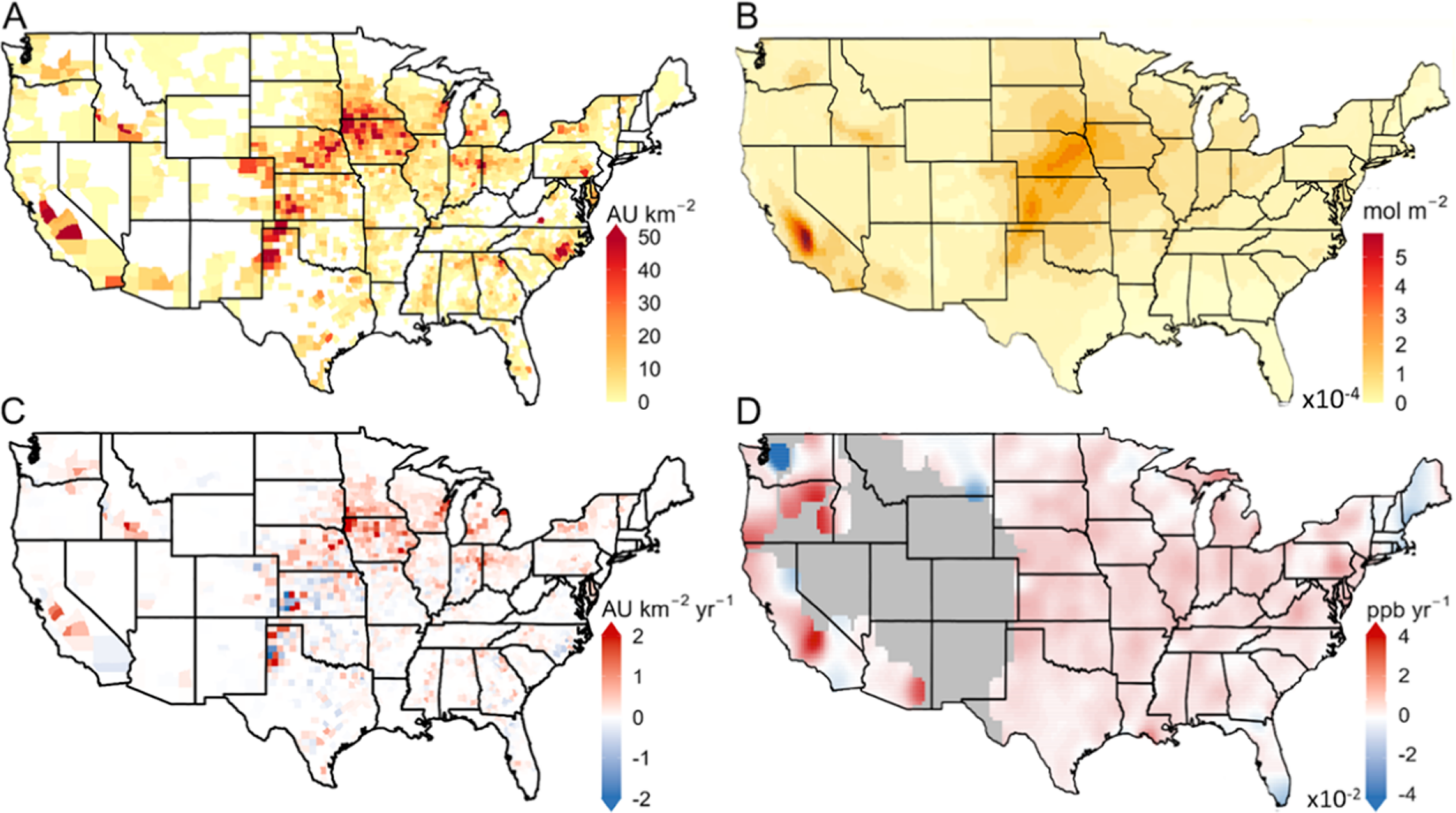
Spatial distributions and trends of animal unit density and ammonia over the CONUS. (A) Animal units (1000 lb. of live weight) at medium- and large-sized CAFOs normalized by county area for the year 2017. (B) Average summertime 2017 NH_3_ mixing ratios from the ESA IASI product. (C) Change in county-level AU density at medium- and large-sized CAFOs from 2002 to 2017. (D) Change in summertime NH_3_ mixing ratio from NASA AIRS product from 2002 to 2017.

**Figure 2:**
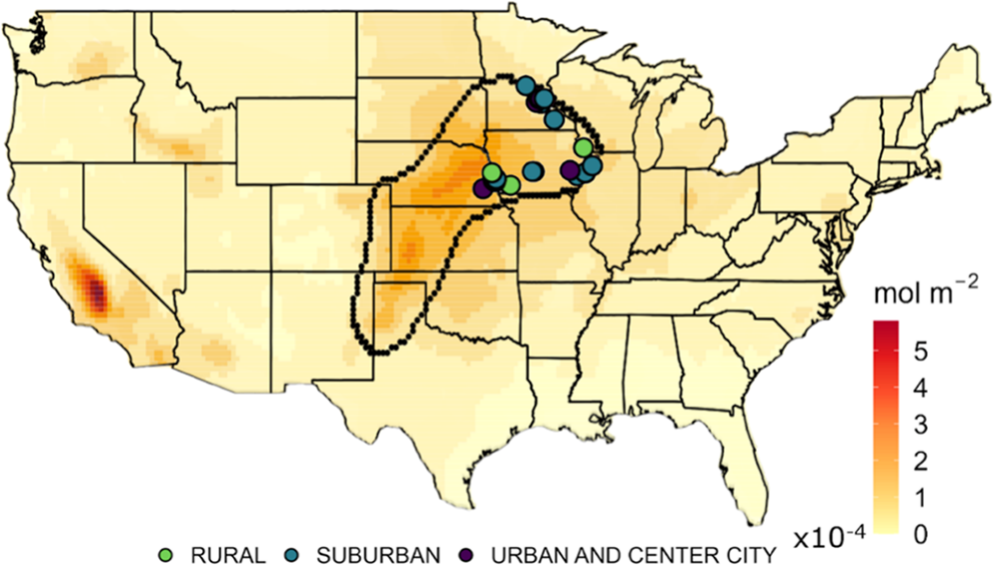
Relationship between remotely detected NH_3_ and EPA surface PM_2.5_ monitor coverage within the “hotspot” region of highest animal unit density.

Spatial patterns in animal unit density at medium and large CAFOs are consistent with satellite-detected NH_3_ concentrations over the CONUS (Figure 1). Ammonia emission rates from livestock vary with the animal type, population size, and farm management. Practices that mitigate air pollution emissions at farms with lower animal densities, such as sustainable application of livestock waste to fields, are less feasible at the industrial scale. For example, feedlot ammonia emissions are much higher than those for pasture-raised animals due, in part, to greater reliance on manure storage and silage.^54^ Livestock waste is the predominant source of controllable ammonia in the U.S., a factor of 3 higher than all other sources combined, including fertilizer use (Figure S1), and some fraction of fertilizer is animal manure. Summertime ammonia from IASI for 2017 indicates the highest values are over the Midwest, Central Valley of California, Arizona, and locations in the Northwest, where 2017 county-level animal unit density derived from the AgCensus is also high (Figure 1a,b and Movie S1). The ammonia spatial pattern is consistent with the animal unit density of feedlot cattle and hogs in the Midwest, dairy cows in the CA Central Valley, and cattle and dairy cows in Arizona and the Northwest (Movies S2–S4). The spatial patterns for the AU density level set derived from USDA’s 2017 AgCensus and IASI-detected ammonia data for 2017 are remarkably similar (Figure 2). Across the CONUS for 2017, there is a significant positive correlation between remotely detected NH_3_ and area-normalized AU density (*r* = 0.642). Ammonia to the east of the level set for animal unit density is consistent with prevailing easterly winds over the Midwest Plains, and suggests application of wind adjustments^53^ at the surface and aloft could improve correlation.

Rising emissions as a consequence of increasing animal consolidation are a plausible explanation for the temporal trends in rising ammonia mixing ratios. Over the past two decades, raw animal numbers rose approximately 6%, while the number of CAFOs rose more quickly, approximately 16% over the last 10 years (Figure S2). Farm consolidation and greater county-level animal density are found for all analyzed animal categories, including cattle, dairy cows, hogs, broiler chickens, and egg-laying hens from 2002 to 2017 (Movies S1–S6). AIRS ammonia data from 2002 to 2017 indicates an increasing atmospheric NH_3_ burden across the CONUS, with higher rates of increase in the Midwest and CA Central Valley (Figure 1d). This pattern is similar to the growth in the number of CAFOs and increasing deposition of reduced nitrogen in agricultural areas.^15,17,55^ Evaluation of EPA emission estimates during intensive observation periods (IOPs) and with IASI data generally find a low bias for ambient ammonia in rural Midwest regions.^56−58^ This is consistent with the low bias in estimates of CAFO-associated animals in publicly available Federal archives. Recent efforts by environmental interest groups (EIGs) and some States have improved CAFO accounting. However, recent application of a deep-learning algorithm to satellite data detects 15% more poultry CAFOs in North Carolina than recorded by EIGs.^59^

Accounting for animal numbers normalized by county area implicitly accounts for some degree of industrial-scale management practices that affect ammonia emissions. For example, CAFO management relies on increased use of silage and storage that results in higher emissions of air pollutants.^60,61^ Measured ammonia emission rates from anaerobic lagoons are substantial,^61,62^ and large dairy and swine CAFOs preferentially employ anaerobic lagoons for sanitary waste management, particularly in the Midwest Plains^61^ where satellites detect ammonia hotspots. In air quality simulations, Hu et al. find that summertime ammonia observations over the Midwest Plains region are up to a factor of 4 higher than model predictions.^56^ The livestock ammonia emissions employed in those air quality simulations are developed from EPA’s NEI.^47^ Evaluation of NEI agricultural emissions finds the highest predictive error for ammonia (90%) is for anaerobic waste lagoons.^62^ In general, ambient NH_3_ is most abundant over rural agricultural land that includes both cropland and CAFOs (Figure S3), and where surface air quality monitoring locations are thinly distributed (Figure 2).

**Figure 3:**
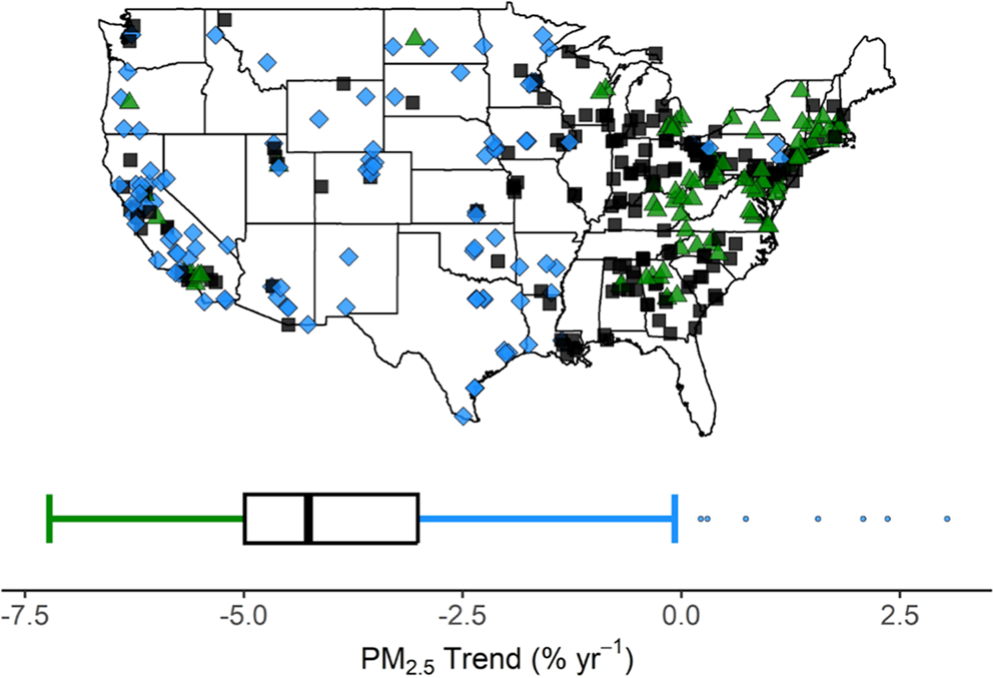
Trends in EPA measured PM_2.5_. Each point represents an EPA PM_2.5_ monitor active over the study period (2002–2017), and is color-coded according to the quartiles of the overall trend distribution, where black points represent trends within the interquartile range, blue points represent trends above the third quartile (less decrease than average), and green points represent trends below the first quartile (greater decrease than average).

Surface monitoring sites are sparsely located in rural agricultural regions, and air quality is more difficult to quantitatively assess. In and near the geographic areas with the largest increases in NH_3_ and highest animal unit density as identified above, trends in PM_2.5_ mass appear to lag behind the national average (Figure 3). Approximately one-third of all EPA air quality sites are located in rural settings, and ∼10% are in agricultural locations (Figure S4). The strongest declines in PM_2.5_ mass are generally observed east of the Mississippi River, particularly in urban areas and downwind of large industrial sources of the Southeast and Ohio Valley, in addition to localized monitoring sites in California (Figure 3). In areas where ammonia mixing ratios are rising and animal unit density is high, other air quality metrics are stagnant or also getting worse. Some specific Midwest surface sites document rising mixing ratios of SO_2_ and NO_2_ (Figures S5 and S6). Nonlinearities in the gas-phase oxidation of SO_2_ and NO_2_, the subsequent gas-to-particle partitioning, aerosol pH, and aerosol liquid water content complicate relationships between PM_2.5_ and precursor emissions.^63^ Within the boundaries of the animal unit level set (Figure 2), there are 23 surface sites active during the study period. Only three sites are classified as rural location settings. The available monitors are primarily located in the northeast corner of the geographic area, far away from satellite-detected ammonia hotspots, and are insufficient to properly assess air quality trends fully representative of the entire region.

**Figure 4:**
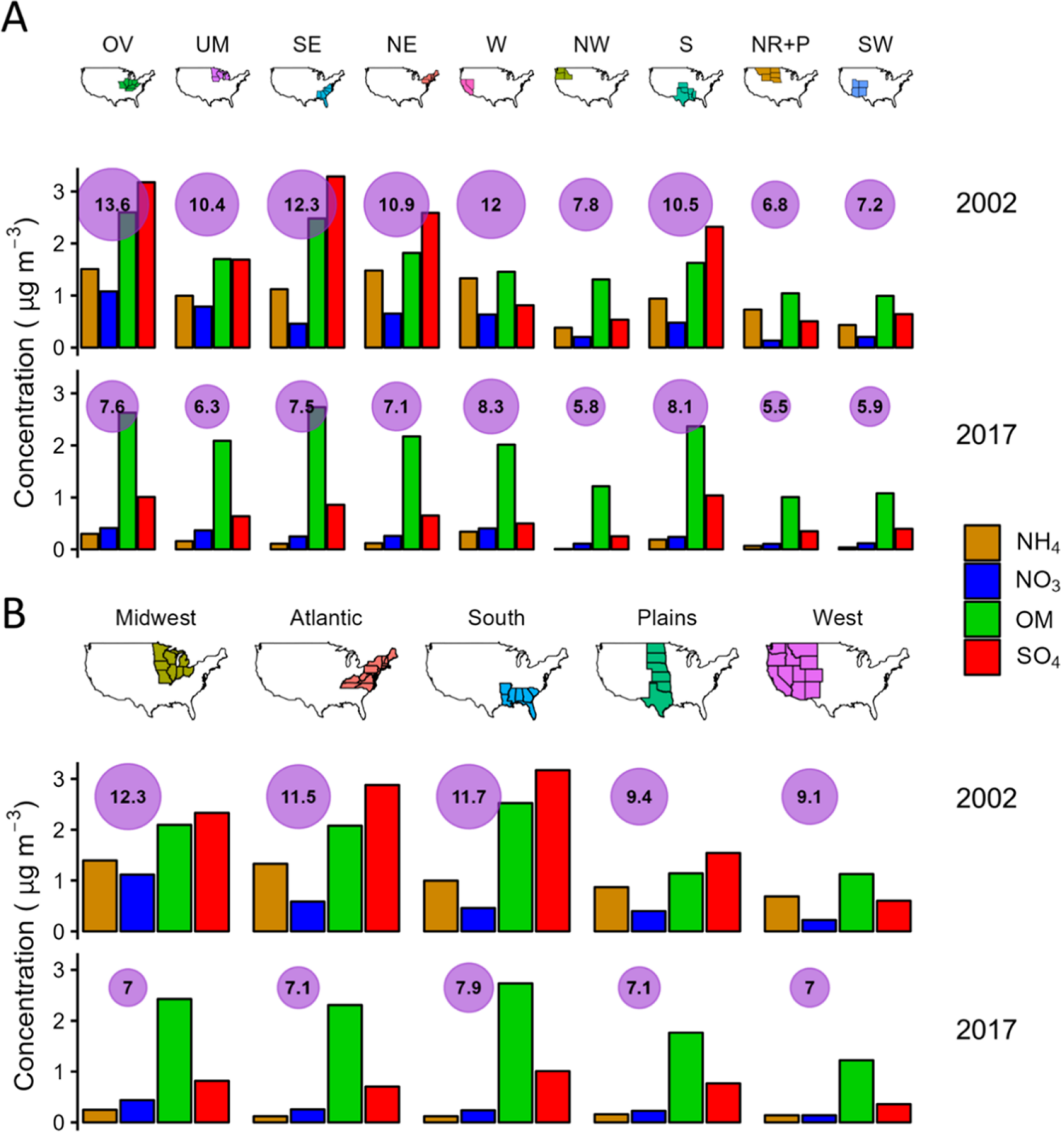
Trends in PM_2.5_ mass and speciation across U.S. EPA climate regions (A) and USDA Farm production regions (B). OV refers to the Ohio Valley, UM refers to the Upper Midwest, SE refers to the Southeast, NE refers to the Northeast, W refers to the West, NW refers to the Northwest, S refers to the South, NR+P refers to the Northern Rockies and Plains, and SW refers to the Southwest. Each column represents a different EPA- or USDA-defined region, mapped in row 1. Row 2 represents average concentrations for each region in 2002, and row 3 shows the same for 2017. Purple circles display the average PM_2.5_ mass for each year and are sized accordingly. Bar charts show annual average concentrations of ammonium (orange), nitrate (blue), organic matter (green), and sulfate (red), measured through EPA CSN.

Conventional federal-level frameworks and modeling tools, such as air quality network sites, their regional definitions, and emission estimates used in assessments, are not ideal to quantify air pollution amount or chemical composition in areas where CAFO emissions are abundant and the air pollution burden is rising. For example, in national and regional assessments, the EPA documents improving surface air quality. However, those assessments have an implicit bias that largely excludes agricultural locations, even for predominantly rural areas. For example, using EPA-defined climate regions, the largest decreases in PM_2.5_ mass from 2002 to 2017 are due to sulfate and occur in the Ohio Valley (66 sites, −44%), Upper Midwest (48 sites, −39%), Southeast (60 sites, −39%), and Northeast (83 sites, −35%) (Figure 4a, Tables S1 and S3). Employing USDA Farm Production Expenditure regions, the largest PM_2.5_ mass concentration decreases (94 sites, −43%) occur in the Midwest region (Figure 4b, Tables S2 and S4) and are due to sulfate. This finding reflects many earlier findings that note the success of acid rain rules that reduce SO_2_ emissions and sulfate formation and is consistent with a primarily urban distribution of Eastern and Midwest monitoring sites (e.g., Detroit, Minneapolis). Such distribution hinders accurate assessment of air quality for the Midwest and other regions that are largely rural and agricultural.

Air pollution policy in the U.S. is primarily focused on large point sources, mobile emissions, and sources of toxic pollutants in highly populated areas. Declines in anthropogenic emissions and improved urban air quality demonstrate the successful implementation of this policy. Yet, most of the U.S. land area is not urban and approximately half is agricultural.^64^ Air quality studies related to health and environmental justice typically focus on urban and suburban areas.^65−67^ Rural PM_2.5_ is toxic^68^ and impacts human and livestock health.^69^ Neglect of agricultural regions in air quality analyses may be inconsistent with national goals for environmental equity.^70^ The agricultural sector relies heavily on a vulnerable immigrant workforce.^71^ In one study, dairy workers in California’s Central Valley, who identify primarily as Hispanic/Latino, experience decreased lung function due to particulate matter exposure in relation to work shift.^72^ As air quality generally improves across the U.S., frontline communities in agricultural areas may be left behind, and a relative dearth of data prevents accurate assessment.

Strategies to reduce agricultural air pollution and its impacts are widely discussed and debated. Because a significant proportion of U.S. meat production is for export, mitigation through changes in trade strategies and production-based abatement (e.g., more efficient manure management and fertilizer use, increasing free-range farming practices) are proposed.^6,13,73^ Consumer-side actions are also predicted to have a substantial influence over agricultural ammonia. Reduction of animal product consumption, red meat especially, and utilization of more nitrogen-efficient protein sources reduce predictions of food-production-related PM_2.5_ and subsequent associated mortality and societal costs.^13,18,73^ Research suggests controls on agricultural ammonia emissions could be a cost-effective strategy to improve air quality and safeguard human health.^9,13,74^ For example, wintertime NH_3_ control strategies that focus on improved farm animal housing and manure management are predicted to be more cost-effective for reducing PM_2.5_ mass than existing control strategies for SO_2_ or NO_*x*_.^9^ However, quantitative assessment of the impacts of such strategies would be difficult given the poor spatiotemporal coverage in air quality networks at rural locations in addition to the lack of full transparency in animal data. There is precedent for satellite tools to correct ammonia seasonality in inventory estimates from the land sector within EPA.^75^ Air quality policies reliant on regulation-defined surface measurements may need to evolve to incorporate advanced tools.

## Supporting Information Available

Trend in county-level animal unit density across all animal types (Movie S1) (AVI)
Trend in county-level feedlot cattle animal unit density (Movie S2) (AVI)
Trend in county-level dairy cow animal unity density (Movie S3) (AVI)
Trend in county-level hog animal unit density (Movie S4) (AVI)
Trend in county-level broiler chicken animal unit density (Movie S5) (AVI)
Trend in county-level layer chicken animal unit density (Movie S6) (AVI)
Information on NH_3_ emissions by sector, trends in animal inventory numbers and CAFO practices, analysis of land use (cropland and CAFO spatial distribution) and EPA AQS monitor location settings, trends in NO_2_ and SO_2_ ambient concentrations (Figures S1–S6), and numeric data for Figure 4 (Tables S1–S4) (PDF)

## Supplementary Material

es3c06947_si_001.avi

es3c06947_si_002.avi

es3c06947_si_003.avi

es3c06947_si_004.avi

es3c06947_si_005.avi

es3c06947_si_006.avi
