# Supplementary material for: Data Gap: Air Quality Networks Miss Air Pollution from Concentrated Animal Feeding Operations
Source: Environ Sci Technol. 2023 Nov 30;57(49):20718–25. doi: 10.1021/acs.est.3c06947 (PMC10720380; doi:10.1021/acs.est.3c06947)
Supplement: Supplementary file 7 — es3c06947_si_007.pdf [file es3c06947_si_007.pdf]

Supporting Information for "The Data Gap: Air Quality Networks Miss Air Pollution from Concentrated Animal Feeding Operations"

Alyssa M. Burns[1], Gabriel Chandler[2], Kira J. Dunham[3,†] and Annmarie G. Carlton[1,]*

[1]Department of Chemistry, University of California, Irvine, CA, 92617, United States

[2]Department of Mathematics and Statistics, Pomona College, Claremont, CA, 91711, United States

[3]Food and Water Watch, Washington, DC, 20036, United States

* Corresponding Author: agcarlton@uci.edu

This supporting information includes the following 6 figures and 4 tables:

Figure S1. 2017 NH_3 emissions by sector.

Figures S2. Animal head counts and NPDES-reported CAFO numbers.

Figure S3. Spatial distribution of cropland cover and CAFO practices.

Figure S4. Location setting and land use for available AQS sites.

Figure S5. Trend in ambient SO_2 concentrations.

Figure S6. Trend in ambient NO_2 concentrations

Table S1. Average $PM_{2.5}$ mass concentrations, standard deviations (SD), and regional site numbers for EPA-defined U.S. climate regions.

Tables S2. Average $PM_{2.5}$ mass concentrations, standard deviations (SD), and regional site numbers for USDA-defined Farm Production regions**.**

Table S3. Average mass concentrations for major $PM_{2.5}$ constituents, standard deviations (SD), and regional site numbers for EPA-defined U.S. climate regions.

Table S4. Average mass concentrations for major $PM_{2.5}$ constituents, standard deviations (SD), and regional site numbers for EPA-defined U.S. climate regions.

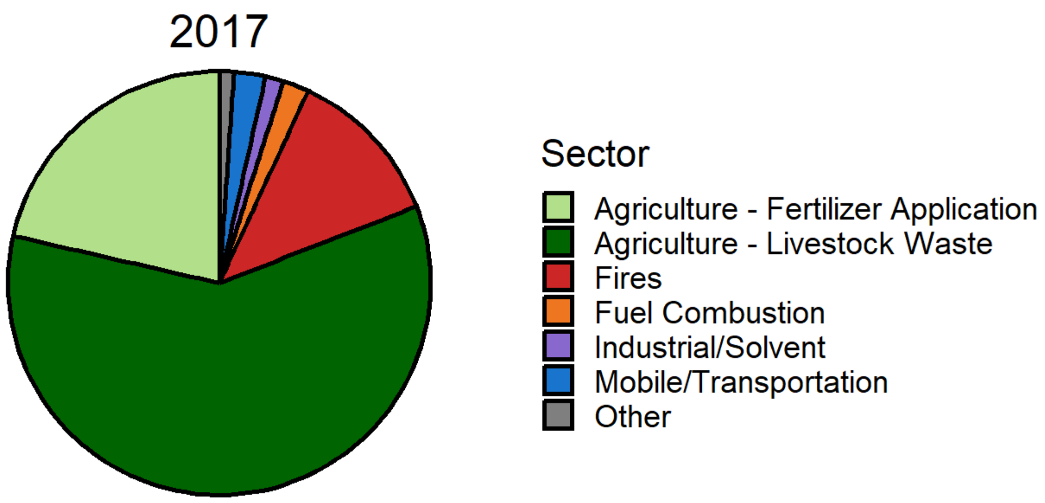

Figure S1. Fractional contribution by source sector to total 2017 annual ammonia emission in the contiguous U.S. according EPA's National Emissions Inventory.

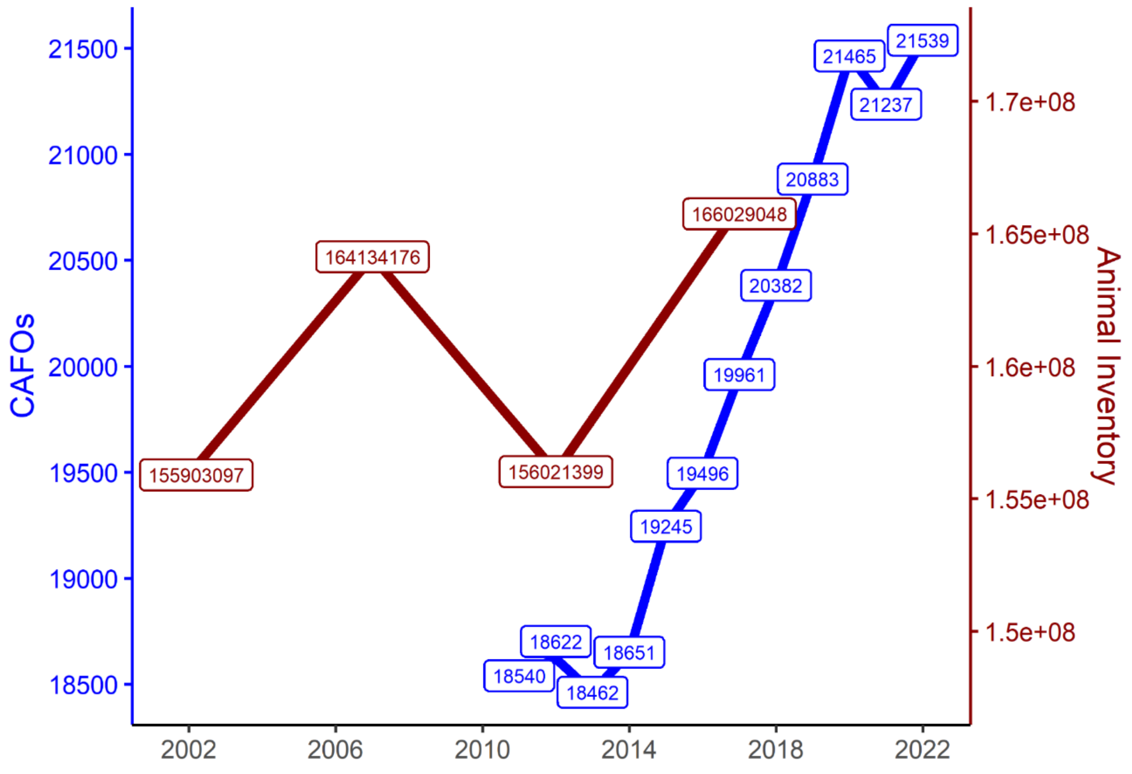

Figure S2. Annual farm animal and CAFO numbers from National Pollution Discharge Elimination System (NPDES) reporting to the EPA. All available State-level data (2011-2022) is used and aggregated across the CONUS. Animal inventory represents USDA AgCensus reported head counts for cows and swine, which are often in open air confinements

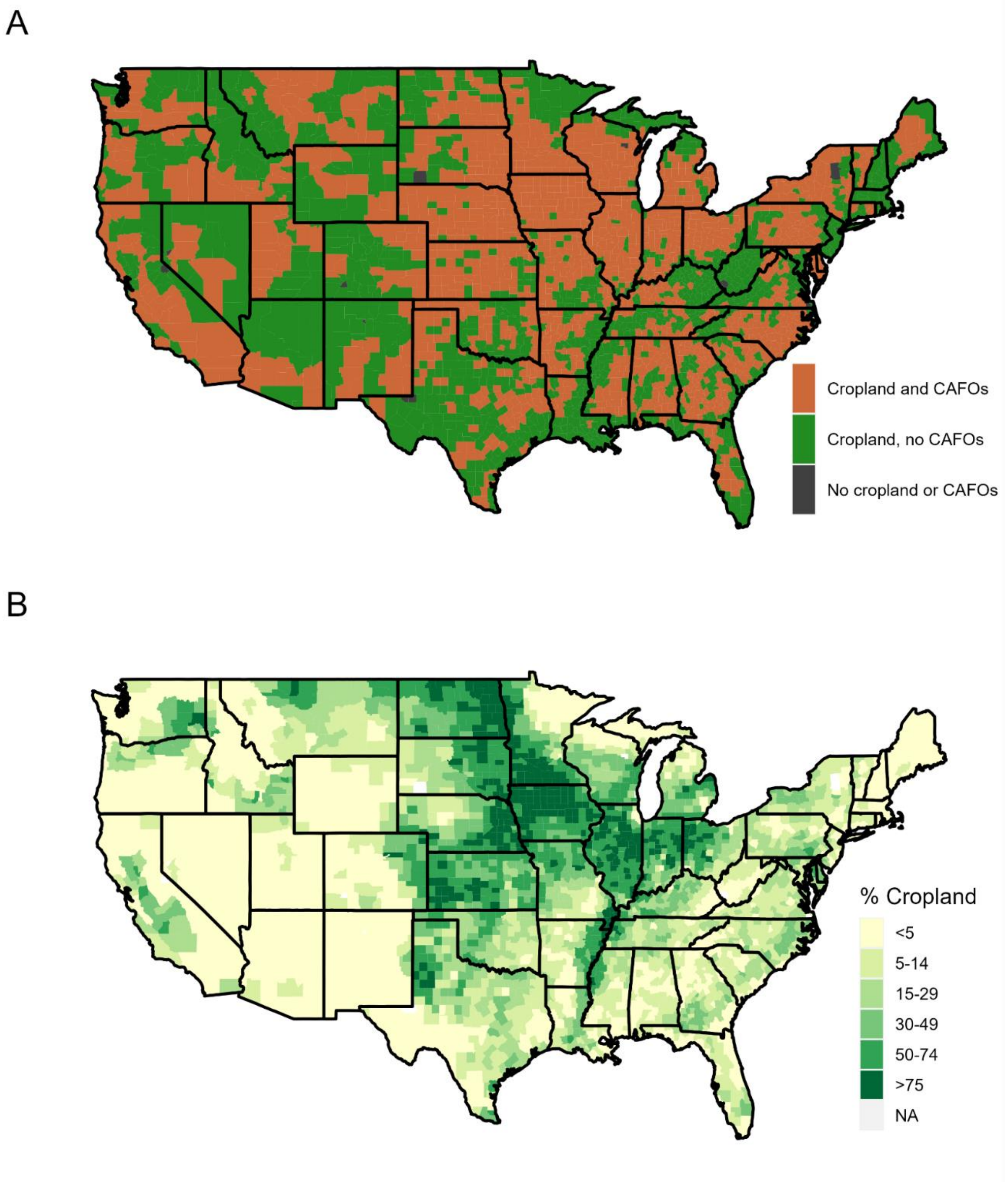

Figure S3. USDA-reported cropland cover and industrial farming practices from the 2017 AgCensus over the CONUS (A). USDA-reported cropland at the county level (B). Regions with cropland and CAFOs are more spatially similar to satellite NH_3 than areas of high cropland cover (no CAFOs).

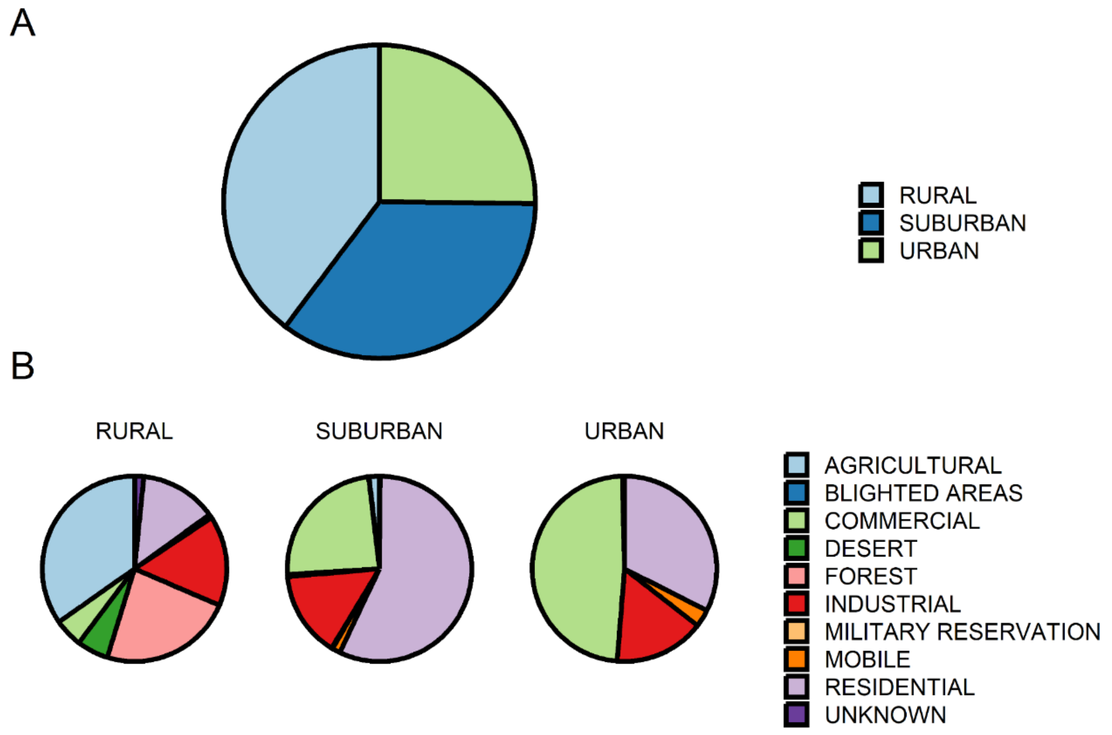

Figure S4. EPA-reported AQS site location setting (A) and site land use (B).

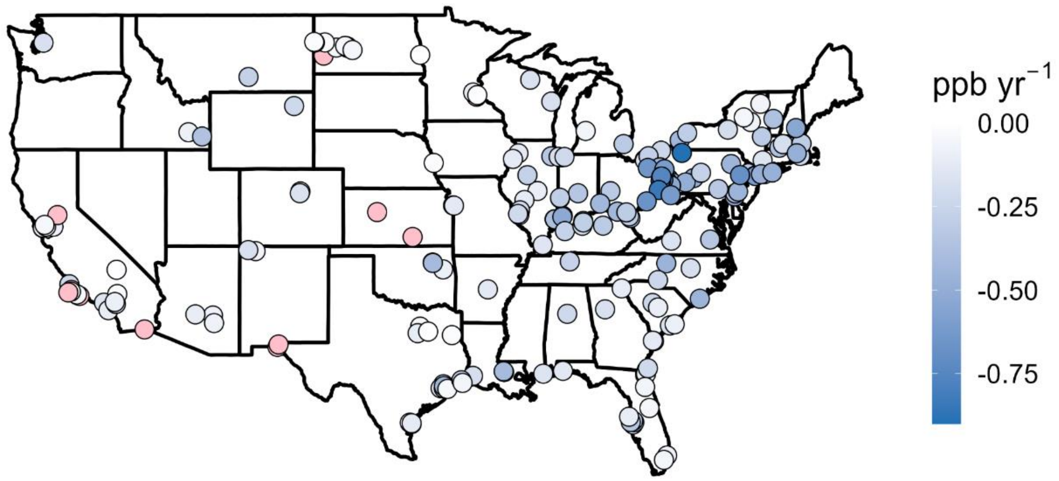

Figure S5. Trends in ambient SO_2 concentrations from 2002-2017. Blue points represent a decreasing trend according to the colorbar. Pink points represent sites that experience a slightly positive trend (<0.1 ppb yr^{-1}).

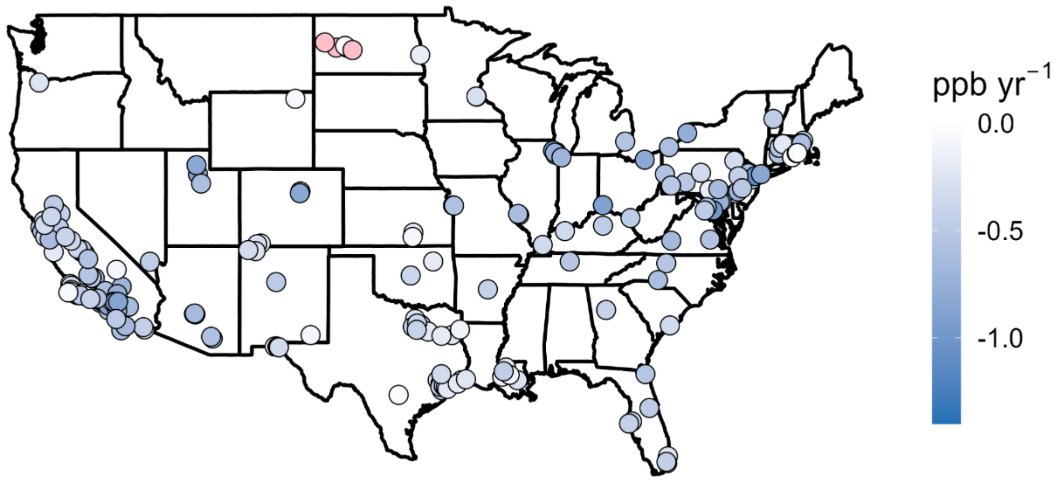

Figure S6. Trends in ambient NO_2 concentrations from 2002-2017. Blue points represent a decreasing trend according to the colorbar. Pink points represent sites that experience a slightly positive trend (<0.05 ppb yr^{-1}).

Table S1. Average $PM_{2.5}$ mass concentrations, standard deviations (SD), and regional site numbers for EPA-defined U.S. climate regions.

EPA Region (sites)	Median 2002 $PM_{2.5}$ ($\mu g\ m^{-3}$)	2002 SD	Median 2017 $PM_{2.5}$ ($\mu g\ m^{-3}$)	2017 SD	% Change
Ohio Valley (66)	13.6	8.2	7.6	4.3	-44.1
Upper Midwest (48)	10.4	7.7	6.3	4.4	-39.4
Southeast (60)	12.3	7.0	7.5	4.1	-38.7
Northeast (83)	10.9	8.6	7.1	4.8	-34.9
West (56)	12.0	13.1	8.3	8.8	-30.9
Northwest (10)	7.8	9.4	5.8	10.5	-25.6
South (34)	10.5	6.2	8.1	4.1	-23.3
Northern Rockies and Plains (18)	6.8	6.5	5.5	6.1	-19.4
Southwest (24)	7.2	7.7	5.9	6.3	-18.0
CONUS (399)	11.0	8.9	7.1	5.9	-35.2

Table S2. Average $PM_{2.5}$ mass concentrations, standard deviations (SD), and regional site numbers for USDA-defined Farm Production Regions

.

USDA Region (sites)	Median 2002 $PM_{2.5}$ ($\mu g\ m^{-3}$)	2002 SD	median 2017 $PM_{2.5}$ ($\mu g\ m^{-3}$)	2017 SD	% Change
Midwest (94)	12.3	8.1	7.0	4.5	-43.2
Atlantic (126)	11.5	8.4	7.1	4.5	-38.3
South (51)	11.7	6.5	7.9	4.2	-32.4
Plains (32)	9.4	6.4	7.1	5.1	-24.5
West (96)	9.1	11.5	7.0	8.3	-23.1
CONUS (399)	11.0	8.9	7.1	5.9	-35.2

Table S3. Average mass concentrations for major $PM_{2.5}$ constituents, standard deviations (SD), and regional site numbers for EPA-defined U.S. climate regions.

EPA Region (total sites)	Chemical Species (sites)	Median 2002 conc. (μg m^{-3})	2002 SD	Median 2017 conc. (μg m^{-3})	2017 SD	% Change
Ohio Valley (32)	SO_4 (32)	3.2	3.2	1.0	0.8	-68.2
	NO_3 (32)	1.1	1.9	0.4	1.4	-62.0
	NH_4 (29)	1.5	1.3	0.3	0.6	-80.1
	OM (26)	2.6	1.6	2.6	2.1	1.2
Upper Midwest (18)	SO_4 (18)	1.7	2.6	0.6	0.6	-62.4
	NO_3 (18)	0.8	2.4	0.4	1.4	-53.5
	NH_4 (14)	1.0	1.4	0.2	0.5	-83.9
	OM (15)	1.7	3.7	2.1	1.9	22.8
Southeast (37)	SO_4 (37)	3.3	2.9	0.9	0.6	-73.9
	NO_3 (36)	0.5	0.7	0.3	0.5	-45.3
	NH_4 (34)	1.1	0.9	0.1	0.2	-90.1
	OM (27)	2.5	3.8	2.7	5.4	10.2
Northeast (31)	SO_4 (31)	2.6	3.8	0.7	0.5	-74.9
	NO_3 (31)	0.7	1.6	0.3	0.9	-60.0
	NH_4 (22)	1.5	1.5	0.1	0.4	-91.9
	OM (29)	1.8	4.4	2.2	1.9	19.5

West (27)	SO_4 (27)	0.8	1.4	0.5	0.6	-38.8
	NO_3 (27)	0.6	6.0	0.4	2.5	-36.3
	NH_4 (12)	1.3	3.1	0.3	1.3	-74.4
	OM (23)	1.5	4.0	2.0	5.7	38.5
Northwest (20)	SO_4 (20)	0.5	0.8	0.3	0.3	-52.5
	NO_3 (20)	0.2	0.8	0.1	0.6	-45.3
	NH_4 (5)	0.4	0.4	0.01	0.1	-97.4
	OM (18)	1.3	8.9	1.2	10.0	-7.0
South (23)	SO_4 (23)	2.3	2.5	1.0	0.9	-55.3
	NO_3 (21)	0.5	1.3	0.2	0.7	-49.6
	NH_4 (17)	0.9	0.9	2.4	0.3	-79.8
	OM (17)	1.6	1.5	0.2	2.5	45.5
Northern Rockies and Plains (22)	SO_4 (22)	0.5	0.9	0.3	0.4	-30.6
	NO_3 (22)	0.1	0.7	0.1	0.5	-19.9
	NH_4 (3)	0.7	0.9	0.1	0.3	-90.4
	OM (22)	1.0	2.2	1.0	13.8	-3.3
Southwest (30)	SO_4 (30)	0.6	0.6	0.4	0.4	-38.3
	NO_3 (30)	0.2	1.7	0.1	1.1	-41.6
	NH_4 (5)	0.4	1.5	0.04	1.0	-90.7
	OM (30)	1.0	4.2	1.1	2.4	8.7

CONUS (240)	SO_4 (240)	1.3	2.7	0.6	0.7	-56.0
	NO_3 (237)	0.4	2.6	0.2	1.3	-45.9
	NH_4 (141)	1.1	1.6	0.2	0.7	-85.6
	OM (207)	1.4	4.8	1.9	6.4	31.6

Table S4. Average mass concentrations for major $PM_{2.5}$ constituents, standard deviations (SD), and regional site numbers for USDA-defined Farm Production Regions.

USDA Region (total sites)	Chemical Species (sites)	median 2002 conc. ($\mu g\ m^{-3}$)	2002 SD	median 2017 conc. ($\mu g\ m^{-3}$)	2017 SD	% Change
Midwest (41)	SO4 (41)	2.3	3.0	0.8	0.8	-64.9
	NO3 (41)	1.1	2.2	0.4	1.5	-60.5
	NH4 (34)	1.4	1.4	0.3	0.5	-82.1
	OM (36)	2.1	3.1	2.4	2.0	15.7
Atlantic (55)	SO4 (55)	2.9	3.6	0.7	0.5	-75.5
	NO3 (55)	0.6	1.4	0.3	0.8	-56.1
	NH4 (46)	1.3	1.3	0.1	0.4	-91.0
	OM (44)	2.1	3.7	2.3	2.0	11.0
South (30)	SO4	3.2	2.7	1.0	0.7	-68.1
	NO3	0.5	0.7	0.2	0.4	-47.9
	NH4	1.0	0.9	0.1	0.3	-88.0

	OM	2.5	4.0	2.7	6.0	8.5
Plains (22)	SO4 (22)	1.5	2.2	0.8	0.8	-50.2
	NO3 (20)	0.4	1.4	0.2	0.7	-42.5
	NH4 (13)	0.9	0.9	0.2	0.3	-81.6
	OM (20)	1.1	1.4	1.8	3.0	55.1
West (92)	SO4 (92)	0.6	0.9	0.4	0.5	-40.3
	NO3 (92)	0.2	3.5	0.1	1.6	-36.8
	NH4 (23)	0.7	2.7	0.1	1.2	-79.7
	OM (86)	1.1	5.5	1.2	9.0	8.8
CONUS (240)	SO4 (240)	1.3	2.7	0.6	0.7	-56.0
	NO3 (237)	0.4	2.6	0.2	1.3	-45.9
	NH4 (141)	1.1	1.6	0.2	0.7	-85.6
	OM (207)	1.4	4.8	1.9	6.4	31.6
